# Aversive anticipations modulate electrocortical correlates of decision-making and reward reversal learning, but not behavioral performance

**DOI:** 10.3389/fnbeh.2022.908454

**Published:** 2022-08-05

**Authors:** Florian Bublatzky, Sabine Schellhaas, Christian Paret

**Affiliations:** ^1^Department of Psychosomatic Medicine and Psychotherapy, Central Institute of Mental Health Mannheim, Medical Faculty Mannheim, Heidelberg University, Mannheim, Germany; ^2^Tel-Aviv Sourasky Medical Center, School of Psychological Sciences, Sagol Brain Institute, Wohl Institute for Advanced Imaging, Tel-Aviv University, Tel Aviv, Israel

**Keywords:** reward reversal learning, threat-of-shock, decision-making, feedback processing, FRN, P3, LPP

## Abstract

Predicting the consequences of one’s own decisions is crucial for organizing future behavior. However, when reward contingencies vary frequently, flexible adaptation of decisions is likely to depend on the situation. We examined the effects of an instructed threat context on choice behavior (i.e., reversal learning) and its electrocortical correlates. In a probabilistic decision-making task, 30 participants had to choose between two options that were either contingent on monetary gains or losses. Reward contingencies were reversed after reaching a probabilistic threshold. Decision-making and reversal learning were examined with two contextual background colors, which were instructed as signals for threat-of-shock or safety. Self-report data confirmed the threat context as more unpleasant, arousing, and threatening relative to safety condition. However, against our expectations, behavioral performance was comparable during the threat and safety conditions (i.e., errors-to-criterion, number of reversal, error rates, and choice times). Regarding electrocortical activity, feedback processing changed throughout the visual processing stream. The feedback-related negativity (FRN) reflected expectancy-driven processing (unexpected vs. congruent losses and gains), and the threat-selective P3 component revealed non-specific discrimination of gains vs. losses. Finally, the late positive potentials (LPP) showed strongly valence-specific processing (unexpected and congruent losses vs. gains). Thus, regardless of contextual threat, early and late cortical activity reflects an attentional shift from expectation- to outcome-based feedback processing. Findings are discussed in terms of reward, threat, and reversal-learning mechanisms with implications for emotion regulation and anxiety disorders.

## Introduction

The consequences of one’s own decisions are a crucial source of information for adjusting future behavior. When the association between choices and outcomes is volatile, the flexible adjustment of decision-making is highly adaptive. Based on trial and error, such reversal learning requires a memory update with the inhibition of an old choice–outcome association and acquisition of a new choice–outcome association. This ability to change previously learned associations is important for adequate social functioning. For instance, Kringelbach and Rolls (2003) showed that participants were readily able to change and reverse their choice behavior based on the facial expressions of others. Changing behavior from one option to another, based on feedback information, involves the anterior cingulate and the medial frontal cortex and modulates face and person perception within fractions of a second (Holroyd and Coles, 2002; Willis et al., 2010; Koban and Pourtois, 2014; Bublatzky et al., 2020a). Moreover, decision-making is no isolated process but occurs within environmental conditions (e.g., social situations) providing additional information about potential rewards or threats. Here, contextual threat or safety can both improve and reduce social cognitive functions such as facial emotion recognition (Bublatzky et al., 2020b; Kavcıoğlu et al., 2021), physiological response priming to emotional stimuli (Costa et al., 2015; Bublatzky et al., 2018), avoidance behavior, and reward reversal learning (Bublatzky et al., 2017; Atlas, 2019; Paret and Bublatzky, 2020).

The anticipation of aversive events is adaptive to avoid harmful situations and foster individual survival. The same is true for potentially rewarding events. Accordingly, humans readily accumulate knowledge about threat and reward contingencies, and aversive/appetitive anticipations are formed even based on very limited information. For instance, the mere verbal instruction about the possibility to receive electric shocks (the “threat-of-shock” paradigm; Grillon et al., 1991) has been shown to provoke a sustained pattern of apprehensions, selective attention to threat cues, and physiological response preparation to cope with the anticipated event (e.g., Bradley et al., 2005; Bublatzky and Schupp, 2012; Robinson et al., 2013). Importantly, participants never experience shocks in this paradigm, and threat associations are based on the mere verbal communication and cognitive representation of threat. Interestingly, such expectation-based processes are surprisingly stable and rather resistant to fast extinction learning (e.g., compared to experiential learning; cf. Bublatzky et al., 2013, 2014, 2022). Since overlooking a threat is far more costly than a false alarm, the absence of shocks is no guarantee that danger has been averted. More specifically, in the absence of contingency experience, the evidence for the (long-term) absence of shocks is much less clear in instructed learning and thus prolongs extinction learning.

Decision-making is based on the hedonic value of behavioral options and their expected outcomes (O’Doherty et al., 2003; Sharot et al., 2009). Here, the decision-maker usually pursues rewarding actions and tends to avoid potentially harmful or disadvantageous consequences (e.g., loss of positive reinforcement), and thus needs to constantly monitor performance and action outcomes. Such outcome evaluation is reflected in the feedback-related negativity (FRN), a negative-going waveform peaking at fronto-central sensor sites about 200–300 ms after relevant feedback presentation (Miltner et al., 1997; Simons, 2010). Based on the reinforcement learning theory (Holroyd and Coles, 2002; Holroyd et al., 2006), the FRN has been suggested to reflect an evaluative signal that varies with action-outcome valence and outcome expectancy. Here, the FRN is particularly pronounced for negative relative to positive outcomes (e.g., losses > gains), and for unexpected relative to expected outcomes (i.e., reflecting a prediction error; Hajcak et al., 2005, 2007; Bellebaum and Daum, 2008; Chase et al., 2011; Walentowska et al., 2016). Based on such feedback information, instrumental learning can flexibly adjust choice behaviors to new environmental stimulus–reward associations (e.g., Atlas, 2019). However, often response conflicts arise between opposing goals, for instance, in the case of anxious avoidance (e.g., costly avoidance; Pittig et al., 2020) or healthy curiosity (e.g., approach despite threat; Schlund et al., 2016; Bublatzky et al., 2017). Thus, similar to threat learning, the expectation of rewards is a powerful mechanism to improve learning and change of behaviors.

In the present study, we examined the temporal dynamics of electrocortical processes underlying decision-making and reversal learning under threatening conditions. To this end, the participants’ task was to choose between two behavioral options (images of apples and oranges, or two female faces) that were associated with either monetary gains or losses. Behavioral options were differentially reinforced and reward contingencies were reversed after reaching a probabilistic threshold of 6–9 correct choices. Importantly, the task was completed within two context conditions in which participants either anticipated aversive electric shocks or were assured to be safe from shocks. Context conditions were indicated by the background color of the screen on which the decision-making task was performed. As in previous research, we predicted that an instructed threat context would be rated as more unpleasant, arousing, and threatening compared to the safety condition (Bublatzky et al., 2010, 2014; Costa et al., 2015). On the neural level, we expected enhanced late positive potentials to instructed threat compared to safety conditions over parieto-occipital visual processing areas as indicators for selective attention and elaborate stimulus processing (Bublatzky and Schupp, 2012; MacNamara and Barley, 2018).

Regarding behavioral performance, we hypothesized detrimental effects of the threatening context on decision-making and reward reversal learning (Bogdan et al., 2010). For instance, in a recent companion study that focused on psychophysiological responding, we observed that threat-of-shock rapidly disrupted reward reversal learning and participants needed more errors to learn new stimulus–reward associations (i.e., errors-to-criterion; Paret and Bublatzky, 2020). With respect to electrocortical indicators, we expected a feedback-related negativity for unexpected relative to expected outcomes (i.e., prediction error) and negative relative to positive outcomes (Bellebaum and Daum, 2008; Chase et al., 2011; Koban and Pourtois, 2014). While these feedback effects have been observed to be reduced in participants with high levels of anxiety (Gu et al., 2010; Aarts and Pourtois, 2012; O’Toole et al., 2012; Voegler et al., 2019), we hypothesized a similar reduction in healthy participants within a threatening context; possibly related to reduced behavioral performance. Moreover, enhanced P3 and LPP amplitudes were expected for negative relative to positive outcomes (e.g., Bellebaum and Daum, 2008; Potts, 2011; Koban and Pourtois, 2014). Here, more pronounced P3 and LPP under threat-of-shock may reflect the threat-increased motivational relevance (e.g., Aarts and Pourtois, 2010, 2012; Bublatzky and Schupp, 2012; Bublatzky et al., 2020a).

## Materials and methods

### Participants

A non-clinical sample of 30 healthy participants (19 females) was recruited from the University of Mannheim (Germany). Age was on average 20.83 years (SD = 1.97) and anxiety and depression questionnaire scores indicate a healthy sample. Specifically, participants were within the normal range in terms of state and trait anxiety (STAI-S and -T; M = 34.60 and 35.07, SD = 8.11 and 8.46; State-Trait Anxiety Inventory; Laux et al., 1981), anxiety sensitivity (ASI; M = 16.8, SD = 7.58; Anxiety Sensitivity Index; Reiss et al., 1986), and depression (BDI II; M = 5.30, SD = 5.78; Beck Depression Inventory; Hautzinger et al., 2009). Moreover, social anxiety was tested using the Social Phobia Inventory (SPIN M = 12.7, SD = 7.12; Stangier and Steffens, 2002), Fear of Negative Evaluation–short scale (FNE-K M = 32.87, SD = 8.60; Reichenberger et al., 2016), and Social Interaction Anxiety Scale (SIAS M = 14.90, SD = 8.81; Stangier et al., 1999).

Participants gave written informed consent to the study protocol and received course credits for participation. Ethical approval was provided by the university’s ethics committee.

### Materials and task

**FIGURE 1:**
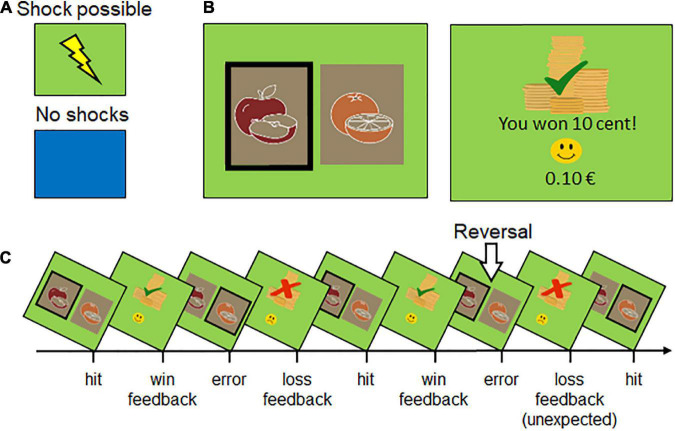
Reward reversal learning as a function of instructed threat or safety. **(A)** Participants are verbally instructed to anticipate up to three unpleasant electric shocks when a specific background color is presented (e.g., green signals threat of shock, blue safety). As a reminder, a print-out of the assigned colors to threat or safety was fixed under the screen and presented on the screen before context conditions changed. **(B)** Participants’ task was to find out and select the rewarded stimulus (i.e., apples vs. oranges, blond vs. dark-haired female face). **(C)** Illustration of reward learning (i.e., apples are rewarded) and reward reversal (i.e., now oranges are rewarded). Errors can be congruent (due to wrong contingency) or incongruent (i.e., unexpected) due to newly changed contingencies.

Neutral face pictures of two female actors selected from the Karolinska Directed Emotional Faces database (KDEF faces “af01nes” and “af19nes”; Lundqvist et al., 1998) and self-drawn pictures of two fruits (apples and oranges) served as stimuli pairs in this study (see Figure 1). Both stimuli together were sized 1680 × 1050 pixels and separately 562 × 762 pixels for each picture. The stimuli were presented on a 22-inch computer screen placed approximately at a distance of 1 m in front of the participants using Presentation software (Neurobehavioral Systems, Berkeley, CA, United States). For each stimulus type (faces and fruits), pictures were visually matched (e.g., similar hair length and picture complexity, as well as drawing style and shape), while maintaining the best possible distinctiveness (e.g., different colors of the hair and fruits).

In four consecutive runs (each two runs with fruits or faces), pictures served as response options and were presented for a maximum of 2 s each (see Figure 1). Within this time, participants had to select one of the stimuli by pressing a left (“N”) or right key (“M”) on a computer keyboard. If no key press was recorded while the picture was shown, the trial was considered a non-response and excluded from the analysis. Choice options were differently reinforced with either a gain or a loss of 0.1 Euro by using a probabilistic learning paradigm (Paret and Bublatzky, 2020). One of the stimuli served as a reward cue with a reinforcement rate of 7:3 (money gain [loss] in 7 [3] out of 10 selections). The other stimulus served as a loss cue, where the participants always lost money if chosen. When a probabilistic learning criterion was met (correct selection of the reward cue 6–9 times in a row), the stimulus-reward contingency was reversed. The total number of reversals depended on how quickly (and how often) the participants learned the new contingencies. Participants were instructed to always choose the picture that was more likely to be rewarded and informed that this reward association may change throughout the experiment. The current balance was always displayed at the bottom of the screen and set to 0 Euro after each run. Participants could not earn any real money in this study.

### Procedure

After the EEG cap and electrodes were attached, participants were seated in a sound-attenuated chamber and were asked to complete questionnaires. Following that, a shock electrode was attached to the inner side of the non-dominant forearm and a brief shock workup was performed to ensure the credibility of the threat instructions (Bublatzky et al., 2010; Riemer et al., 2015) using a Digitimer DS7A stimulator (Digitimer Ltd., Welwyn Garden City, United Kingdom). To this end, the intensity of the electrical shocks was adjusted individually until the stimulation was reported as “maximal unpleasant but not yet painful,” never exceeding a maximum of 10 mA. Participants were then verbally instructed that they had to expect a maximum of three shocks while viewing a specific background color (e.g., green serving as a threat signal), but no shocks would be administered during a safety background (e.g., blue signaling the absence of shocks). The assignment of colors to threat or safety was counterbalanced across participants. Because we focused on the impact of aversive apprehensions (but not experiences) on reversal learning, no shocks were administered during the rest of the experiment (cf. Bublatzky et al., 2014, 2022). Participants were explicitly told that their performance in the reward reversal task was not related to the likelihood of receiving a shock.

Then, the experiment started with alternating runs showing either fruit or face stimuli. Stimulus presentation (e.g., apples and oranges) on the right and left sides of the screen was semi-randomized; the same stimulus was never located on the same side in more than two consecutive trials.

There were 100 trials per experimental run. Each run began with 20 initial trials and a gray background color serving as a control baseline period to allow an initial acquisition of reward contingencies (not analyzed). Following that, two threat and two safety blocks were indicated by green or blue background colors and alternated every 20 trials (counterbalanced order), amounting to a total of 160 trials per threat and safety condition. Each trial began with the presentation of a fixation cross (550 ± 300 ms), followed by the presentation of the picture pair (2000 ± 300 ms) and by a blank screen (800 ± 300 ms), and finally, the feedback presentation showing whether 10 cents were lost or won (1050 ± 300 ms). The frequency of reversals within each condition depended on the subject’s choice behavior in this self-paced learning experiment.

After the last run, participants rated each stimulus individually and together with each of the three possible background colors (blue, green, and gray as control colors) with regard to valence and arousal using the Self-Assessment Manikin (SAM; Bradley and Lang, 1994) and perceived threat on a visual analog Likert-scale [ranging from “not at all” (0) to “very” (10) threatening]. Finally, the participants were debriefed.

### Data recording and reduction

The EEG was recorded using an actiCap system (BrainProducts, Munich, Germany) with 64 channels. Ag/AgCl active electrodes were attached to an elastic cap with a 10-10 electrode placement (Falk Minow Services, Herrsching, Germany). The EEG was recorded continuously at a sampling rate of 500 Hz with FCz as a reference electrode. Data were filtered online from 0.1 to 100 Hz using Vision Recorder Acquisition Software and BrainAmp DC Amplifiers (BrainProducts, Munich, Germany). The impedance of all electrodes was kept below 10 kΩ. The data were analyzed offline with Brain Vision Analyzer 2.0 (BrainProducts), including a low-pass filter at 30 Hz, artifact detection, baseline correction (−100 ms pre-stimulus until 0 ms), and conversion to an averaged reference. Stimulus synchronized epochs were extracted, which lasted from 200 ms before to 800 ms after the stimulus and feedback onset. Separate averaged waveforms were calculated for Context (threat, safety) and Feedback type (win, loss) for each sensor and each participant.

### Data analysis

Data, syntax, and result files can be retrieved here: https://osf.io/ren4v/?view_only=79b4014ef49c46d28acbbb2619e08934.

#### Self-report data

Perceived threat, valence, and arousal were analyzed using repeated measures variance analyses (ANOVA) including the factors Context (threat vs. safety) and Stimulus (faces vs. fruits).

#### Behavioral data

The choice time for correct stimulus selections was analyzed. Performance on the learning task was quantified as the total number of reversals achieved by the participants. Moreover, the percentage of incorrect stimulus selection (%errors = [number of errors * 100]/total number of decisions [i.e., errors and hits]) and the number of errors needed to reach the learning criterion (errors-to-criterion to initiate a contingency reversal) were computed. All performance measures were analyzed using repeated measures variance analyses (ANOVA) with the factors Context (threat vs. safety) and Stimulus (faces vs. fruits). Pearson correlations were used to test associations between performance measures.

**FIGURE 2:**
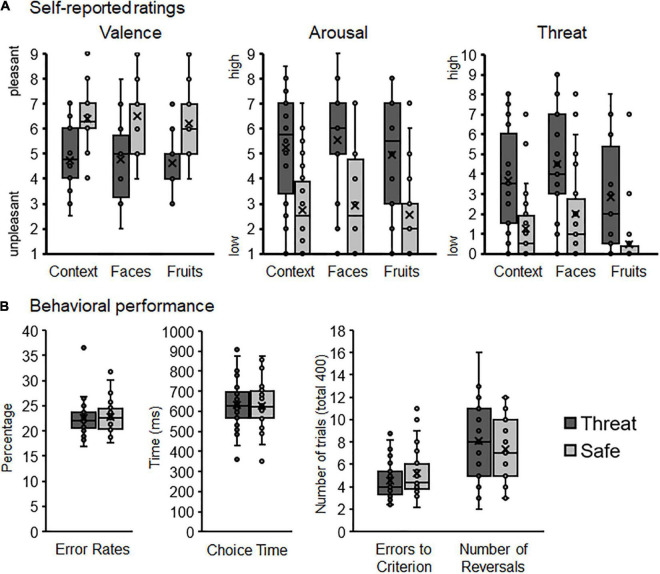
Rating and behavioral data illustrated with boxplots (median, first and third quartiles, and outliers). **(A)** Self-reported valence, arousal, and threat ratings for context conditions, faces, and fruits. **(B)** Behavioral performance measures illustrating error rates, choice times, errors to reach learning criterion, and number of reversals as a function of contextual threat and safety.

Some behavioral variables did not meet the normal distribution criteria for all conditions. While simulation studies have shown that repeated-measures ANOVA is relatively robust to violations of the normal distribution assumption (Vasey and Thayer, 1987; Berkovits et al., 2000), however, we conducted supplementary analyses to confirm our statistical approach. To this end, boxplots were used for visual illustration and exploration of the rating and behavioral data (see Figure 2). These plots show the mean, median, first and third quartiles, minimum and maximum values, as well as outliers (i.e., more than 1.5 times of the interquartile range). Exploratory analyses were performed excluding the identified behavioral outliers. Moreover, non-parametric Friedman Tests were conducted. Importantly, both supplementary strategies confirm our ANOVA findings.

#### Event-related potentials data

As a manipulation check, initial analyses addressed electrocortical processing locked to the onset of the picture and threat-of-shock manipulation using the factors Stimulus (faces vs. fruits) and Context (threat vs. safety). Similar to previous research (Bublatzky et al., 2020a; Schindler and Bublatzky, 2020), the face-selective N170 and threat-associated LPP components were scored over parieto-occipital sensors (PO7 and PO8) between 140 and 180 ms (N170) and between 450 and 750 ms (LPP).

Regarding feedback processing, visual inspection of the waveforms served to identify and localize the relevant ERP components for the factors Context (threat vs. safety), Feedback type (win vs. loss), Stimulus (faces vs. fruits), and Laterality (left vs. right). The feedback-related negativity and P3 component were scored over fronto-central sensors (FC1 and FC2) between 250 and 300 ms (FRN) and between 300 and 400 ms (P3) after feedback onset. Moreover, modulation of Late Positive Potential (LPP) was examined over centro-parietal sensors (CP1 and CP2) between 400 and 600 ms after feedback onset.

For reasons of exploration and illustration, Low-Resolution Electromagnetic Tomography (LORETA; Pascual-Marqui et al., 1994) source estimations were carried out using the inbuilt function of the Brain Vision Analyzer 2.0 (BrainProducts). To this end, current neural sources were estimated from surface ERP differences in feedback processing (unexpected loss-win, unexpected loss-loss, and loss-win) for the FRN, P3, and LPP components, indicating the best match of neuronal sources involved in these comparisons.

Where necessary, Greenhouse Geisser correction was applied and Bonferroni correction was used for multiple comparisons for all dependent variables. As a measure of effect size, the partial η square (η_*p*_^2^) is reported.

## Results

### Self-report ratings

As expected the instructed threat context was rated as more unpleasant, arousing, and threatening compared to the safety condition, Ts(29) = −4.99, 6.25, and 6.95, *ps* < 0.001 (see Figure 2A).

Regarding stimulus ratings, stimuli were rated as more unpleasant, arousing, and threatening when presented in contextual threat compared to safety condition, Context *Fs*_(1,29)_ = 24.87, 39.12, and 48.32, *ps* < 0.001, η_*p*_^2^ = 0.46, 0.57, and 0.63. Moreover, face pictures were rated as more arousing and threatening compared to fruits, Stimulus *Fs*_(1,29)_ = 10.36 and 20.68, *ps* < 0.003 and 0.001, η_*p*_^2^ = 0.26 and 0.42, but no difference was observed regarding valence ratings, *F*_(1,29)_ = 0.89, *p* = 0.35, η_*p*_^2^ = 0.03. No interactions for Stimulus × Context emerged for valence, arousal, or threat ratings, *Fs*_(1,29)_ = 0.41, 0.45, and 0.17, *ps* = 0.53, 0.51, and 0.69, η_*p*_^2^ = 0.01, 0.02, and 0.01.

### Behavioral performance

On average, participants achieved 18.2 ± 6.59 reversals throughout the experiment (minimum of 7, maximum of 32 reversals altogether; cf. Figure 2B). Choice time was 631.69 ms ± 117.89 (CT; 359.12, 865.96), the average percentage of errors was 22.52 ± 2.87 (%errors; 18.75, 32.64), and participants needed 4.52 ± 1.57 errors to reach the learning criterion (i.e., errors-to-criterion; 2.23, 8.6). While %errors and errors-to-criterion are both error rate measures, the total number of reversals achieved during the experiment correlated significantly with errors-to-criterion (*r* = −0.868, *p* < 0.001), but not with %errors (*r* = −0.289, *p* = 0.12). Thus, similar to previous research (Paret and Bublatzky, 2020), errors-to-criterion was the better measure for learning accuracy and was further used for hypothesis testing.

The total number of reversals did neither vary as a function of Stimulus, *F*_(1,29)_ = 2.79, *p* = 0.11, η_*p*_^2^ = 0.09, nor Context, *F*_(1,29)_ = 2.52, *p* = 0.12, η_*p*_^2^ = 0.08, nor Stimulus × Context, *F*_(1,29)_ = 0.42, *p* = 0.52, η_*p*_^2^ = 0.01. Regarding errors-to-criterion, neither the main effects Context, *F*_(1,27)_ = 2.84, *p* = 0.10, η_*p*_^2^ = 0.10, nor Stimulus *F*_(1,27)_ = 4.03, *p* = 0.055, η_*p*_^2^ = 0.13, nor the interaction Stimulus × Context reached significance, *F*_(1,27)_ = 0.19, *p* = 0.67, η_*p*_^2^ = 0.01. When excluding outliers, however, a significant main effect of Stimulus emerged, *F*_(1,24)_ = 4.46, *p* = 0.045, η_*p*_^2^ = 0.16, indicating that participants needed less errors-to-criterion when the task was performed with faces compared to fruit stimuli.

### Electrocortical processing

#### Manipulation check: Face and context processing

**FIGURE 3:**
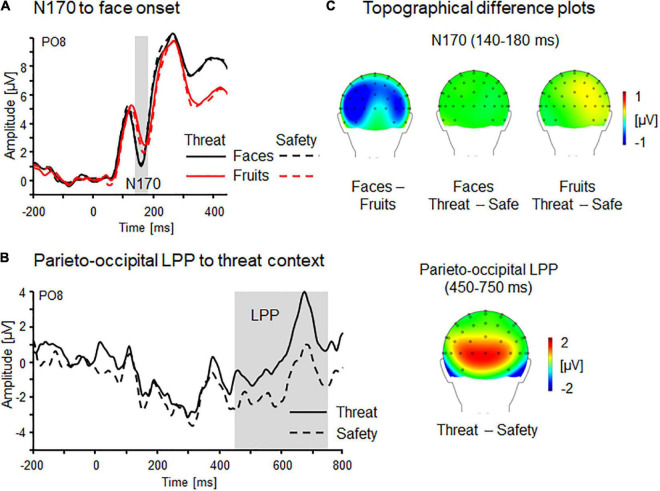
Face and context processing indicated by N170 and LPP components confirm experimental manipulation. **(A)** Event-related brain potential waveforms as a function of stimulus onset (faces, fruits) and context (threat, safety) as revealed by parieto-occipital N170 component (PO8) and **(B)** Parieto-occipital LPP effects linked to the onset of contextual threat and safety. **(C)** Topographical difference plots (faces–fruits and threat–safety for faces and fruits) displaying N170 stimulus effects, and LPP effects linked to the onset of contextual threat or safety. Waveform differences are displayed on the back view of a model head.

Confirming previous research, the face-selective N170 component (PO7/8; 140–180 ms) was found to be more pronounced for faces relative to non-face stimuli (i.e., fruit pictures), Stimulus *F*_(1,28)_ = 17.1, *p* < 0.001, η_*p*_^2^ = 0.38 (see Figure 3A). While no main effects of Context or Laterality emerged, *Fs*_(1,28)_ = 0.13 and 0.09, *ps* = 0.72 and 0.77, η_*p*_^2^ = 0.01 and <0.01, however, a significant three-way interaction Context × Stimulus × Laterality was found, *F*_(1,28)_ = 5.43, *p* < 0.05, η_*p*_^2^ = 0.16. *Post hoc* comparisons showed descriptively more pronounced negativity for fruits during threat compared to safety, *p* = 0.12; also the other comparisons were non-significant, *ps* > 0.60.

Each experimental run started with an initial instruction slide serving as an onset for sustained threat/safety conditions. Although only four trials per participant contribute to these context onset analyses, a threat-enhanced positivity was still observable over parieto-occipital sensor sites (PO7/8; 450–750 ms), Context *F*_(1,29)_ = 7.04, *p* < 0.05, η_*p*_^2^ = 0.20 (see Figure 3B). No main effect of Laterality *F*_(1,29)_ = 3.19, *p* = 0.09, η_*p*_^2^ = 0.10, nor an interaction Context × Laterality was observed, *F*_(1,29)_ = 0.26, *p* = 0.62, η_*p*_^2^ = 0.01.

#### Feedback-related processing: FRN, P3, and LPP

**FIGURE 4:**
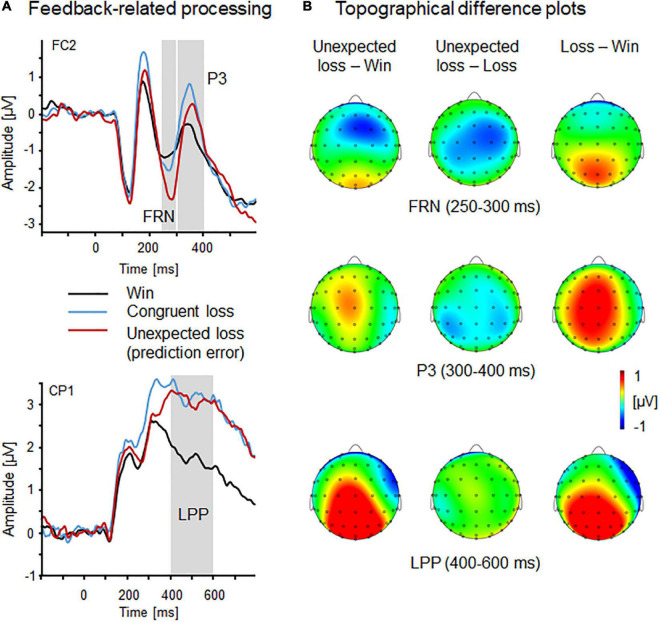
Feedback-related processing indicated by FRN, P3, and LPP components. **(A)** Event-related brain potential waveforms as a function of feedback type (win, congruent loss, and incongruent loss) revealed by fronto-central FRN and P3 (FC2) and centro-parietal LPP (CP1). **(B)** Topographical difference plots (incongruent loss—win, incongruent loss—loss, and loss—win) displaying feedback type effects for the FRN, P3, and LPP components. Waveform differences are displayed on the top view of a model head.

Amplitudes of the feedback-related negativity (FC1/2, 250–300 ms) varied as a function of Feedback Type, *F*_(2,56)_ = 8.67, *p* = 0.001, η_*p*_^2^ = 0.24 (see Figure 4), showing most pronounced negativity for incongruent (unexpected) losses compared to win and congruent loss feedback, *ps* < 0.01, which did not differ from each other, *p* = 1.0. The FRN did not significantly vary between the right compared to the left hemisphere, Laterality, *F*_(1,28)_ = 3.17, *p* = 0.09, η_*p*_^2^ = 0.1. No further main or interaction effect reached significance, Fs < 2.98, *p* > 0.10, η_*p*_^2^ < 0.10.

Regarding the fronto-central P3 component (FC1/2, 300–400 ms), a significant main effect of Feedback Type emerged, *F*_(2,56)_ = 5.45, *p* < 0.01, η_*p*_^2^ = 0.16, indicating that congruent losses were associated with more positive P3 amplitudes compared to win feedback, *p* < 0.05, the other *post hoc* comparisons were not significant, *ps* > 0.26. Moreover, P3 amplitudes were reduced for feedback during threat compared to safety Context, *F*_(1,28)_ = 6.31, *p* < 0.05, η_*p*_^2^ = 0.18. No further main or interaction effect reached significance, Fs < 3.41, *ps* > 0.08, η_*p*_^2^ < 0.11.

Late positive potentials (CP1/2, 400–600 ms) were observed to vary with Feedback Type, *F*_(2,56)_ = 19.94, *p* < 0.001, η_*p*_^2^ = 0.42, showing pronounced positivities to congruent and incongruent losses relative to win feedback, *ps* < 0.001, while losses did not differ from each other, *p* = 0.64. Moreover, LPP were more positive for fruit stimuli compared to faces, *F*_(1,28)_ = 7.03, *p* < 0.05, η_*p*_^2^ = 0.20. No further main or interaction effects reached significance, Fs < 2.32, *ps* > 0.14, η_*p*_^2^ < 0.08.

## Discussion

The present study examined the impact of anticipated threat or safety on decision-making and reward reversal learning. Self-report data confirmed the threat context as more unpleasant, arousing, and threatening relative to safety condition; this was also reflected in threat-enhanced late positive potentials (parieto-occipital LPP, 450–750 ms) to the onset of context conditions. Contrary to our expectations, behavioral parameters during the threat and safety conditions were comparable (e.g., errors-to-criterion, number of reversal), and even after excluding outliers, the contextual threat did not significantly modulate decision-making. Regarding feedback processing, electrocortical indicators of error monitoring and reward reversal learning were observed. Specifically, a pronounced feedback-related negativity (fronto-central FRN, 250–300 ms) was found for unexpected losses compared to both congruent losses and win feedback. The fronto-central P3 component (300–400 ms) discriminated between congruent loss and gain feedback and was sensitive to contextual conditions. P3 amplitude was reduced during threat compared to safety context. Finally, the late positive potential to feedback cues (centro-parietal LPP, 400–600 ms) was more pronounced for loss information (congruent and unexpected) compared to gains. Overall, feedback processing changed throughout the visual processing stream—from expectancy-driven (FRN: unexpected vs. congruent losses and gains) to indifferent discrimination of gains and losses (P3 also threat-selective) and to valence-specific processing (LPP: unexpected and congruent losses vs. gains). Thus, regardless of contextual threat, early and late cortical activity reflects an attentional shift from expectation- to outcome-based feedback processing.

Information about the outcome of actions is central to performance control in decision-making tasks, especially in potentially threatening conditions. The FRN is tied to external outcome feedback (e.g., gain or loss) and presumably reflects the selective processing of negative performance outcomes and varies with prediction error (Miltner et al., 1997; Gehring and Willoughby, 2002; Hajcak et al., 2005, 2007). Confirming previous research, we observed pronounced FRN in response to unexpected negative outcomes relative to win feedback (e.g., Holroyd and Coles, 2002; Holroyd et al., 2006; Yasuda et al., 2004). Building upon this, the present study adds that the FRN was not affected by contextual threat or safety, pointing to separate systems involved in feedback and threat processing. However, it must be noted that the feedback options (monetary gains or losses) were not diagnostic of the threat-of-shock signals (i.e., background colors). In this regard, the relevance of contextual and situational settings is an important aspect of the process of decision-making and performance monitoring (e.g., Bublatzky et al., 2017; Pittig et al., 2018). For instance, in an experimental setting where participants were under social observation, increased FRN amplitudes were found in socially anxious individuals under observation compared to a non-social control condition (Voegler et al., 2019). Situational aspects can thus alter the processing of feedback, especially if they are relevant to the decision task at hand (Koban and Pourtois, 2014). Future research may further address the role of situational aspects (e.g., perceived stress and threat) and performance monitoring for social behaviors (e.g., avoidance, stockpiling, or donation behavior; Walentowska et al., 2016; Pittig et al., 2020).

A non-interactive pattern of contextual threat and feedback processing continued for the P3 component, which was shown to respond to feedback valence, risk level of decisions, attentional allocation, and memory update processes (Yeung and Sanfey, 2004; Willis et al., 2010; Schuermann et al., 2012; Bublatzky et al., 2020a). Extending these results, the present study shows that the P3 was sensitive to both the valence of feedback and contextual threat, suggesting simultaneous but independent processing of threat and feedback at this stage. Specifically, enhanced P3 amplitudes were observed for congruent loss relative to win feedback, which actually reflects the most relevant information for reversal learning because negative feedback indicates the need for updating choice–outcome associations. Alternatively, this finding may relate to the probability of the (less often) negative relative to the (more often) positive feedback (Walentowska et al., 2016). Speaking against this explanation, the least often incongruent loss feedback did not reveal significantly enhanced P3 amplitudes. Moreover, reduced feedback-related P3 amplitudes during a threatening situation may reflect interference effects of contextual threat on attentional allocation and memory processes (Schindler and Bublatzky, 2020). Here, recent research observed the frontal P3 component as sensitive to reversal learning and memory update processes. For instance, P3 modulations were reported when participants were cued to switch associations formed with angry faces (Willis et al., 2010) and for angry-looking face identities that changed their meaning from safety to cueing threat (Bublatzky et al., 2020a).

Finally, parieto-occipital late positive potentials (LPP) showed a clear valence-specific and outcome-oriented processing pattern (i.e., in/congruent losses vs. gains). Here, the motivational relevance of negative feedback indicates the need to adjust behavior, irrespective of contextual settings. Consistent with this reasoning, numerous previous studies showed a particularly pronounced LPP amplitude for negative information [for reviews see Schupp et al. (2006) and Schindler and Bublatzky (2020)], which has been associated with defensive motivational states, action-related processing, and psychophysiological response priming (e.g., withdrawal or avoidance behaviors; Löw et al., 2008; Bublatzky and Schupp, 2012; Ming et al., 2021). Faithful to this motivational relevance, threat-enhanced LPP amplitudes were observed only at the onset of the threat context but not at the onset of feedback (which was not threat-predictive) within a threatening context.

**FIGURE 5:**
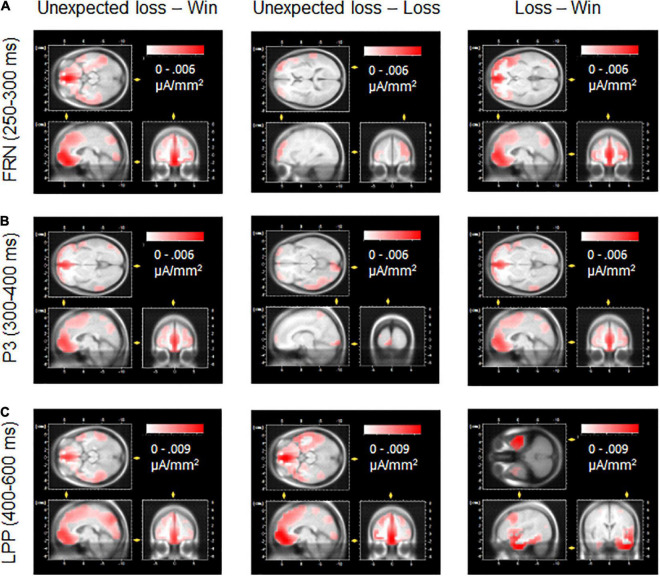
LORETA source estimations for the differential effects of feedback types as revealed by the FRN, P3, and LPP components. The medial frontal gyrus and anterior cingulate emerged as relevant sources for the FRN **(A)** and P3 **(B)** components discriminating negative compared to positive feedback. **(C)** For the LPP, frontal lobe and orbital gyrus best-matched the differential cortical activity to unexpected loss vs. loss and win feedback. For the LPP difference loss vs. win, the best source match referred to the temporal lobe and inferior temporal gyrus.

Taken together, these results support the notion that the FRN functionally reflects a mechanism that evaluates whether an outcome meets the expectations (Miltner et al., 1997; Simons, 2010; Potts, 2011; Koban and Pourtois, 2014). However, the P3 and later LPP components are more sensitive to outcome evaluation that is dependent on both motivational aspects and more top-down controlled processing (e.g., attentional allocation and memory update processes; Yeung and Sanfey, 2004; Wu and Zhou, 2009; Bublatzky et al., 2020a; Ming et al., 2021). Regarding the neural structures involved, LORETA source estimations of FRN, P3, and LPP feedback effects confirm previous imaging research (see Figure 5) that identified the anterior cingulate cortex and medial frontal gyrus as key structures involved in behavior monitoring and feedback processing (e.g., Gehring and Willoughby, 2002; Kringelbach and Rolls, 2003; Potts, 2011; Gläscher et al., 2012). Interestingly, a clear source discrimination between congruent and incongruent losses was found only for the LPP, suggesting the frontal lobe and orbital gyrus as the best match for differential cortical activity. Here, future imaging research needs to clarify the interplay of neural structures and temporal dynamics involved in congruence and feedback processing, as well as its relevance for decision-making (Cunningham et al., 2004; Frömer et al., 2019; Bublatzky et al., 2020b).

Regarding the effects of threat and stress on behavioral performance, previous research has shown mixed results depending on, for example, threat manipulation, behavioral tasks, and individual anxiety disposition [for reviews see Starcke and Brand (2012) and Robinson et al. (2013)]. However, in the few studies that addressed reward learning, reduced reward learning was observed under threat-of-shock (Bogdan and Pizzagalli, 2006; Bogdan et al., 2010). For instance, our direct partner study focusing on psychophysiological response measures (Paret and Bublatzky, 2020) observed that more errors-to-criterion were needed under threat (relative to safety) but no difference emerged for the overall number of reached reversals using the same experimental task as in the present study. The present behavioral data only replicate the null findings regarding the total number of reversals, but no reduced learning of reward reversal during threat (i.e., more errors-to-criterion) was found. Diverging findings may relate to several factors, e.g., the composition of the study samples (e.g., female vs. mixed female/male; e.g., Villanueva-Moya and Expósito, 2021) in combination with the stimulus materials (fruits and female faces). However, other aspects such as reward and incentive values, choice conflicts, and exploration behavior are of particular interest to follow-up on (e.g., Clark et al., 2012; Endrass et al., 2013; Bublatzky et al., 2017; Pittig et al., 2018).

Another noteworthy aspect regards the divergent result patterns for self-reported ratings, behavioral performance, and electrocortical measures. While ratings and ERP findings clearly confirm previous threat-of-shock studies (e.g., pronounced threat ratings and threat-enhanced parieto-occipital LPP amplitudes), there was no contextual threat effect for any of the behavioral parameters examined (i.e., errors-to-criterion, number of reversal, error rates, and choice time), even when using non-parametric tests or removing behavioral outliers. From a conceptual perspective, however, it is not entirely surprising that anticipatory threat effects do not reach the level of overt behavioral change. For instance, it is a well-known phenomenon that different levels of emotion measurements do not necessarily coincide (e.g., Lang, 1993; Lang et al., 1997; Ventura-Bort et al., 2022). Moreover, with regard to anticipatory aversive states, several recent studies have observed threat effects at the neural level, but no behavioral equivalents, for example, in the recognition of facial identities, subtle facial expressions, or objects within emotional scenes (Bublatzky et al., 2020b; Schellhaas et al., 2020; Ventura-Bort et al., 2020; Arnold et al., 2021). Here, aversive anticipations can influence perceptual and attentional processes (i.e., “not missing the perception of a potential threat”), but without reaching the threshold that triggers (costly) activation of physiological response systems or even overt behavioral responses (e.g., Schellhaas et al., 2022). Future research may use other threat manipulations (e.g., anticipation vs. experience of pain or relief) to examine the combined impact of severe aversive conditions on reward learning (e.g., in pain-related disorders; Al-Obaidi et al., 2000; Leknes et al., 2011; Seidel et al., 2015).

Although the present healthy sample does not allow conclusions to be drawn regarding psychological disorders and clinical interventions, the current design addresses and combines important mechanisms (i.e., reward-, reversal-, and threat learning) that are critically involved in the development and maintenance of psychopathology. Future research may address the clinical relevance in samples with high impulsivity, difficulties in emotion regulation, and stress- and trauma-related disorders (e.g., attention deficit hyperactivity disorder and borderline personality disorder; e.g., Hirsh and Inzlicht, 2008; Endrass et al., 2013; Weinberg et al., 2015, 2016; Paret et al., 2017; Bellato et al., 2021).

In summary, the present study examined reward-reversal learning under threatening or safe contextual settings. Key findings confirm successful threat manipulation through verbal threat and safety learning, which led to enhanced threat, unpleasantness, and arousal ratings. While no impact of contextual threat was found for behavioral parameters, electrocortical processing revealed threat-selective P3 amplitudes and late positive potentials. Regardless of the contextual threat, a change from early expectancy-driven (feedback-related negativity) to valence-specific processing (late positive potentials) was observed. Future research needs to address the (mal-)adaptive impact of stress and threat on feedback processing in more complex decision-making situations in healthy and clinical populations.

## Data availability statement

The data, syntax, and result files presented in the study are publicly available. This data can be found here: https://osf.io/ren4v/?view_only=79b4014ef49c46d28acbbb2619e08934.

## Ethics statement

The studies involving human participants were reviewed and approved by Central Institute of Mental Health/Medical Faculty Mannheim, University of Heidelberg. The patients/participants provided their written informed consent to participate in this study.

## Author contributions

FB designed the study, performed data analyses, wrote and revised the manuscript, and acquired funding. SS collected data, was involved in data analyses, and revised the manuscript. CP designed the study, programmed the experimental procedure, was involved in data analyses, and revised the manuscript. All authors contributed to the article and approved the submitted version.

## References

[B1] Aarts K. Pourtois G. (2010). Anxiety not only increases, but also alters early error-monitoring functions. *Cogn. Affect. Behav. Neurosci.* 10 479–492. 21098809 10.3758/CABN.10.4.479

[B2] Aarts K. Pourtois G. (2012). Anxiety disrupts the evaluative component of performance monitoring: an ERP study. *Neuropsychologia* 50 1286–1296. 10.1016/j.neuropsychologia.2012.02.012 22374184

[B3] Al-Obaidi S. M. Nelson R. M. Al-Awadhi S. Al-Shuwaie N. (2000). The role of anticipation and fear of pain in the persistence of avoidance behavior in patients with chronic low back pain. *Spine* 25 1126–1131. 10.1097/00007632-200005010-00014 10788858

[B4] Arnold N. R. González Cruz H. Schellhaas S. Bublatzky F. (2021). A multinomial modelling approach to face identity recognition during instructed threat. *Cogn. Emot.* 35 1302–1319. 10.1080/02699931.2021.1951175 34253158

[B5] Atlas L. Y. (2019). How instructions shape aversive learning: higher order knowledge, reversal learning, and the role of the amygdala. *Curr. Opin. Behav. Sci.* 26 121–129.

[B6] Bellato A. Norman L. Idrees I. Ogawa C. Y. Waitt A. Zuccolo P. F. (2021). A systematic review and meta-analysis of altered electrophysiological markers of performance monitoring in Obsessive-Compulsive Disorder (OCD), Gilles de la Tourette Syndrome (GTS), Attention-Deficit/Hyperactivity Disorder (ADHD) and Autism. *Neurosci. Biobehav. Rev.* 131 964–987. 10.1016/j.neubiorev.2021.10.018 34687698

[B7] Bellebaum C. Daum I. (2008). Learning-related changes in reward expectancy are reflected in the feedback-related negativity. *Eur. J. Neurosci.* 27 1823–1835. 18380674 10.1111/j.1460-9568.2008.06138.x

[B8] Berkovits I. Hancock G. R. Nevitt J. (2000). Bootstrap Resampling Approaches for Repeated Measure Designs: relative Robustness to Sphericity and Normality Violations. *Educ. Psychol. Meas.* 60 877–892.

[B9] Bogdan R. Pizzagalli D. A. (2006). Acute stress reduces reward responsiveness: implications for depression. *Biol. Psychiatry* 60 1147–1154. 16806107 10.1016/j.biopsych.2006.03.037 PMC2288705

[B10] Bogdan R. Perlis R. H. Fagerness J. Pizzagalli D. A. (2010). The impact of mineralocorticoid receptor ISO/VAL genotype (rs5522) and stress on reward learning. *Genes Brain Behav.* 9 658–667. 10.1111/j.1601-183X.2010.00600.x 20528958 PMC2921022

[B11] Bradley M. M. Lang P. J. (1994). Measuring emotion: the self-assessment manikin and the semantic differential. *J. Behav. Ther. Exp. Psychiatry* 25 49–59. 7962581 10.1016/0005-7916(94)90063-9

[B12] Bradley M. M. Moulder B. Lang P. J. (2005). When good things go bad: the reflex physiology of defense. *Psychol. Sci.* 16 468–473. 10.1111/j.0956-7976.2005.01558.x 15943673

[B13] Bublatzky F. Alpers G. W. Pittig A. (2017). From avoidance to approach: the influence of threat-of-shock on reward-based decision making. *Behav. Res. Ther.* 96 47–56. 10.1016/j.brat.2017.01.003 28108010

[B14] Bublatzky F. Schupp H. T. (2012). Pictures cueing threat: brain dynamics in viewing explicitly instructed danger cues. *Soc. Cogn. Affect. Neurosci.* 7 611–622. 10.1093/scan/nsr032 21719425 PMC3427861

[B15] Bublatzky F. Flaisch T. Stockburger J. Schmälzle R. Schupp H. T. (2010). The interaction of anticipatory anxiety and emotional picture processing: an event-related brain potential study. *Psychophysiology* 47 687–696. 10.1111/j.1469-8986.2010.00966.x 20136732

[B16] Bublatzky F. Gerdes A. B. Alpers G. W. (2014). The persistence of socially instructed threat: two threat-of-shock studies. *Psychophysiology* 51 1005–1014. 10.1111/psyp.12251 24942368

[B17] Bublatzky F. Guerra P. M. Pastor M. C. Schupp H. T. Vila J. (2013). Additive effects of threat-of-shock and picture valence on startle reflex modulation. *PLoS One* 8:e54003. 10.1371/journal.pone.0054003 23342060 PMC3546963

[B18] Bublatzky F. Guerra P. Alpers G. W. (2018). Verbal instructions override the meaning of facial expressions. *Sci. Rep.* 8:14988. 10.1038/s41598-018-33269-2 30301956 PMC6177419

[B19] Bublatzky F. Guerra P. Alpers G. W. (2020a). Watch out, he’s dangerous! Electrocortical indicators of selective visual attention to allegedly threatening persons. *Cortex* 131 164–178. 10.1016/j.cortex.2020.07.009 32866901

[B20] Bublatzky F. Kavcıoğlu F. Guerra P. Doll S. Junghöfer M. (2020b). Contextual information resolves uncertainty about ambiguous facial emotions: behavioral and magnetoencephalographic correlates. *NeuroImage* 215:116814. 10.1016/j.neuroimage.2020.116814 32276073

[B21] Bublatzky F. Schellhaas S. Guerra P. (2022). The mere sight of loved ones does not inhibit psychophysiological defense mechanisms when threatened. *Sci. Rep.* 12:2515. 10.1038/s41598-022-06514-y 35169193 PMC8847570

[B22] Chase H. W. Swainson R. Durham L. Benham L. Cools R. (2011). Feedback-related negativity codes prediction error but not behavioral adjustment during probabilistic reversal learning. *J. Cogn. Neurosci.* 23 936–946. 20146610 10.1162/jocn.2010.21456

[B23] Clark L. Li R. Wright C. M. Rome F. Fairchild G. Dunn B. D. (2012). Risk-avoidant decision making increased by threat of electric shock. *Psychophysiology* 49 1436–1443. 10.1111/j.1469-8986.2012.01454.x 22913418

[B24] Costa V. D. Bradley M. M. Lang P. J. (2015). From threat to safety: instructed reversal of defensive reactions. *Psychophysiology* 52 325–332. 25250656 10.1111/psyp.12359

[B25] Cunningham W. A. Raye C. L. Johnson M. K. (2004). Implicit and explicit evaluation: fMRI correlates of valence, emotional intensity, and control in the processing of attitudes. *J. Cogn. Neurosci.* 16 1717–1729. 10.1162/0898929042947919 15701224

[B26] Endrass T. Koehne S. Riesel A. Kathmann N. (2013). Neural correlates of feedback processing in obsessive–compulsive disorder. *J. Abnorm. Psychol.* 122:387. 10.1037/a0031496 23421527

[B27] Frömer R. Dean Wolf C. K. Shenhav A. (2019). Goal congruency dominates reward value in accounting for behavioral and neural correlates of value-based decision-making. *Nat. Commun.* 10:4926. 10.1038/s41467-019-12931-x 31664035 PMC6820735

[B28] Gehring W. J. Willoughby A. R. (2002). The medial frontal cortex and the rapid processing of monetary gains and losses. *Science* 295 2279–2282. 10.1126/science.1066893 11910116

[B29] Gläscher J. Adolphs R. Damasio H. Bechara A. Rudrauf D. Calamia M. (2012). Lesion mapping of cognitive control and value-based decision making in the prefrontal cortex. *Proc. Natl. Acad. Sci. U. S. A.* 109 14681–14686. 22908286 10.1073/pnas.1206608109 PMC3437894

[B30] Grillon C. Ameli R. Woods S. W. Merikangas K. Davis M. (1991). Fear-potentiated startle in humans: effects of anticipatory anxiety on the acoustic blink reflex. *Psychophysiology* 28 588–595. 1758934 10.1111/j.1469-8986.1991.tb01999.x

[B31] Gu R. Ge Y. Jiang Y. Luo Y. J. (2010). Anxiety and outcome evaluation: the good, the bad and the ambiguous. *Biol. Psychol.* 85 200–206. 10.1016/j.biopsycho.2010.07.001 20619312 PMC4041009

[B32] Hajcak G. Holroyd C. B. Moser J. S. Simons R. F. (2005). Brain potentials associated with expected and unexpected good and bad outcomes. *Psychophysiology* 42 161–170. 10.1111/j.1469-8986.2005.00278.x 15787853

[B33] Hajcak G. Moser J. S. Holroyd C. B. Simons R. F. (2007). It’s worse than you thought: the feedback negativity and violations of reward prediction in gambling tasks. *Psychophysiology* 44 905–912. 10.1111/j.1469-8986.2007.00567.x 17666029

[B34] Hautzinger M. Keller F. Kühner C. Beck A. T. (2009). *Beck Depressions-Inventar: BDI II; Manual.* Frankfurt: Pearson Assessment.

[B35] Hirsh J. B. Inzlicht M. (2008). The devil you know: neuroticism predicts neural response to uncertainty. *Psychol. Sci.* 19 962–967. 10.1111/j.1467-9280.2008.02183.x 19000202

[B36] Holroyd C. B. Coles M. G. H. (2002). The neural basis of human error processing: reinforcement learning, dopamine, and the error-related negativity. *Psychol. Rev.* 109 679–709. 12374324 10.1037/0033-295X.109.4.679

[B37] Holroyd C. B. Hajcak G. Larsen J. T. (2006). The good, the bad and the neutral: electrophysiological responses to feedback stimuli. *Brain Res.* 1105 93–101. 10.1016/j.brainres.2005.12.015 16427615

[B38] Kavcıoğlu F. C. Bublatzky F. Pittig A. Alpers G. W. (2021). Instructed threat enhances threat perception in faces. *Emotion* 21:419. 10.1037/emo0000708 31829719

[B39] Koban L. Pourtois G. (2014). Brain systems underlying the affective and social monitoring of actions: an integrative review. *Neurosci. Biobehav. Rev.* 46 71–84. 10.1016/j.neubiorev.2014.02.014 24681006

[B40] Kringelbach M. L. Rolls E. T. (2003). Neural correlates of rapid reversal learning in a simple model of human social interaction. *Neuroimage* 20 1371–1383. 10.1016/S1053-8119(03)00393-8 14568506

[B41] Lang P. J. (1993). “The three-system approach to emotion,” in *The Structure of Emotion*, eds Birbaumer N. Öhman A. (Göttingen: Hogrefe and Huber), 18–30.

[B42] Lang P. J. Bradley M. M. Cuthbert B. N. (1997). Motivated attention: affect, activation, and action. *Attent. Orient.* 97:135.

[B43] Laux L. Glanzmann P. Schaffner P. Spielberger C. D. (1981). *Das State-Trait-Angstinventar [The State-Trait Anxiety Inventory].* Göttingen: Hogrefe.

[B44] Leknes S. Lee M. Berna C. Andersson J. Tracey I. (2011). Relief as a reward: hedonic and neural responses to safety from pain. *PLoS One* 6:e17870. 10.1371/journal.pone.0017870 21490964 PMC3072382

[B45] Löw A. Lang P. J. Smith J. C. Bradley M. M. (2008). Both predator and prey: emotional arousal in threat and reward. *Psychol. Sci.* 19 865–873. 18947351 10.1111/j.1467-9280.2008.02170.x PMC3612950

[B46] Lundqvist D. Flykt A. Öhman A. (1998). *Karolinska Directed Emotional Faces—KDEF (CD ROM).* Stockholm: Karolinska Institute.

[B47] MacNamara A. Barley B. (2018). Event-related potentials to threat of predictable and unpredictable shock. *Psychophysiology* 55:e13206. 10.1111/psyp.13206 PMC6150785 30112760

[B48] Miltner W. H. Braun C. H. Coles M. G. (1997). Event-related brain potentials following incorrect feedback in a time-estimation task: evidence for a “generic” neural system for error detection. *J. Cogn. Neurosci.* 9 788–798. 10.1162/jocn.1997.9.6.788 23964600

[B49] Ming X. Lou Y. Zou L. Lei Y. Li H. Li Y. (2021). The cumulative effect of positive and negative feedback on emotional experience. *Psychophysiology* 58:e13935. 10.1111/psyp.13935 34459511

[B50] O’Toole S. A. Weinborn M. Fox A. M. (2012). Performance monitoring among non-patients with obsessive–compulsive symptoms: ERP evidence of aberrant feedback monitoring. *Biol. Psychol.* 91 221–228. 10.1016/j.biopsycho.2012.06.005 22749966

[B51] O’Doherty J. P. Dayan P. Friston K. Critchley H. Dolan R. J. (2003). Temporal difference models and reward-related learning in the human brain. *Neuron* 38 329–337. 12718865 10.1016/s0896-6273(03)00169-7

[B52] Paret C. Bublatzky F. (2020). Threat rapidly disrupts reward reversal learning. *Behav. Res. Ther.* 131:103636. 10.1016/j.brat.2020.103636 32387886

[B53] Paret C. Jennen-Steinmetz C. Schmahl C. (2017). Disadvantageous decision-making in borderline personality disorder: partial support from a meta-analytic review. *Neurosci. Biobehav. Rev.* 72 301–309. 10.1016/j.neubiorev.2016.11.019 27914943

[B54] Pascual-Marqui R. D. Michel C. M. Lehmann D. (1994). Low resolution electromagnetic tomography: a new method for localizing electrical activity in the brain. *Int. J. Psychophysiol.* 18 49–65. 7876038 10.1016/0167-8760(84)90014-x

[B55] Pittig A. Hengen K. Bublatzky F. Alpers G. W. (2018). Social and monetary incentives counteract fear-driven avoidance: evidence from approach-avoidance decisions. *J. Behav. Ther. Exp. Psychiatry* 60 69–77. 10.1016/j.jbtep.2018.04.002 29747141

[B56] Pittig A. Wong A. H. Glück V. M. Boschet J. M. (2020). Avoidance and its bi-directional relationship with conditioned fear: mechanisms, moderators, and clinical implications. *Behav. Res. Ther.* 126:103550. 10.1016/j.brat.2020.103550 31981801

[B57] Potts G. F. (2011). Impact of reward and punishment motivation on behavior monitoring as indexed by the error-related negativity. *Int. J. Psychophysiol.* 81 324–331. 10.1016/j.ijpsycho.2011.07.020 21855583 PMC3195890

[B58] Reichenberger J. Schwarz M. Koenig D. Wilhelm F. H. Voderholzer U. Hillert A. (2016). Translation of the Brief Fear of Negative Evaluation Scale-Revised (BFNE-R) and Validation of the German Version (FNE-K). *Diagnostica* 62 169–181.

[B59] Reiss S. Peterson R. A. Gursky D. M. McNally R. J. (1986). Anxiety sensitivity, anxiety frequency and the prediction of fearfulness. *Behav. Res. Ther.* 24 1–8. 3947307 10.1016/0005-7967(86)90143-9

[B60] Riemer M. Bublatzky F. Trojan J. Alpers G. W. (2015). Defensive activation during the rubber hand illusion: ownership versus proprioceptive drift. *Biol. Psychol.* 109 86–92. 10.1016/j.biopsycho.2015.04.011 25960069

[B61] Robinson O. J. Vytal K. Cornwell B. R. Grillon C. (2013). The impact of anxiety upon cognition: perspectives from human threat of shock studies. *Front. Hum. Neurosci.* 7:203. 10.3389/fnhum.2013.00203 23730279 PMC3656338

[B62] Schellhaas S. Arnold N. Schmahl C. Bublatzky F. (2020). Contextual source information modulates neural face processing in the absence of conscious recognition: a threat-of-shock study. *Neurobiol. Learn. Mem.* 174:107280. 10.1016/j.nlm.2020.107280 32702504

[B63] Schellhaas S. Schmahl C. Bublatzky F. (2022). Incidental learning of faces during threat: no evidence for increased autonomic arousal to “unrecognized” threat identities. *Res. Square* [Preprint]. 10.21203/rs.3.rs-1619038/v1 37832817

[B64] Schindler S. Bublatzky F. (2020). Attention and emotion: an integrative review of emotional face processing as a function of attention. *Cortex* 130 362–386. 32745728 10.1016/j.cortex.2020.06.010

[B65] Schlund M. W. Brewer A. T. Magee S. K. Richman D. M. Solomon S. Ludlum M. (2016). The tipping point: value differences and parallel dorsal–ventral frontal circuits gating human approach–avoidance behavior. *Neuroimage* 136 94–105. 10.1016/j.neuroimage.2016.04.070 27153979

[B66] Schuermann B. Endrass T. Kathmann N. (2012). Neural correlates of feedback processing in decision-making under risk. *Front. Hum. Neurosci.* 6:204. 10.3389/fnhum.2012.00204 22783182 PMC3390593

[B67] Schupp H. T. Flaisch T. Stockburger J. Junghöfer M. (2006). Emotion and attention: event-related brain potential studies. *Prog. Brain Res.* 156 31–51. 17015073 10.1016/S0079-6123(06)56002-9

[B68] Seidel E. M. Pfabigan D. M. Hahn A. Sladky R. Grahl A. Paul K. (2015). Uncertainty during pain anticipation: the adaptive value of preparatory processes. *Hum. Brain Mapp.* 36 744–755. 10.1002/hbm.22661 25324216 PMC6869185

[B69] Sharot T. De Martino B. Dolan R. J. (2009). How choice reveals and shapes expected hedonic outcome. *J. Neurosci.* 29 3760–3765. 10.1523/JNEUROSCI.4972-08.2009 19321772 PMC2675705

[B70] Simons R. F. (2010). The way of our errors: theme and variations. *Psychophysiology* 47 1–14. 19929897 10.1111/j.1469-8986.2009.00929.x

[B71] Stangier U. Steffens M. (2002). *). Social Phobia Inventory (SPIN)–Deutsche Fassung.* Frankfurt am Main: Psychologisches Institut der Universität Frankfurt am Main.

[B72] Stangier U. Heidenreich T. Berardi A. Golbs U. Hoyer J. (1999). Die erfassung sozialer phobie durch social interaction anxiety scale (SIAS) und die social phobia scale [Assessment of social phobia by the Social Interaction Anxiety Scale (SIAS) and the Social Phobia Scale (SPS)]. *Zeitschrift für Klinische Psychol.* 28 28–36. 10.1026/0084-5345.28.1.28

[B73] Starcke K. Brand M. (2012). Decision making under stress: a selective review. *Neurosci. Biobehav. Rev.* 36 1228–1248. 10.1016/j.neubiorev.2012.02.003 22342781

[B74] Vasey M. W. Thayer J. F. (1987). The Continuing Problem of False Positives in Repeated Measures ANOVA in Psychophysiology: a Multivariate Solution. *Psychophysiology* 24 479–486. 10.1111/j.1469-8986.1987.tb00324.x 3615759

[B75] Ventura-Bort C. Dolcos F. Wendt J. Wirkner J. Hamm A. O. Weymar M. (2020). Item and source memory for emotional associates is mediated by different retrieval processes. *Neuropsychologia* 145:106606. 10.1016/j.neuropsychologia.2017.12.015 29246488

[B76] Ventura-Bort C. Wendt J. Weymar M. (2022). New insights on the correspondence between subjective affective experience and physiological responses from representational similarity analysis. *Psychophysiology* 11:e14088. 10.1111/psyp.14088 35543530

[B77] Villanueva-Moya L. Expósito F. (2021). Gender differences in decision-making: the effects of gender stereotype threat moderated by sensitivity to punishment and fear of negative evaluation. *J. Behav. Decis. Mak.* 34 706–717.

[B78] Voegler R. Peterburs J. Bellebaum C. Straube T. (2019). Modulation of feedback processing by social context in social anxiety disorder (SAD)–an event-related potentials (ERPs) study. *Sci. Rep.* 9:4795. 10.1038/s41598-019-41268-0 30886233 PMC6423138

[B79] Walentowska W. Moors A. Paul K. Pourtois G. (2016). Goal relevance influences performance monitoring at the level of the FRN and P3 components. *Psychophysiology* 53 1020–1033. 27091565 10.1111/psyp.12651

[B80] Weinberg A. Kotov R. Proudfit G. H. (2015). Neural indicators of error processing in generalized anxiety disorder, obsessive-compulsive disorder, and major depressive disorder. *J. Abnorm. Psychol.* 124:172. 10.1037/abn0000019 25384068

[B81] Weinberg A. Meyer A. Hale-Rude E. Perlman G. Kotov R. Klein D. N. (2016). Error-related negativity (ERN) and sustained threat: conceptual framework and empirical evaluation in an adolescent sample. *Psychophysiology* 53 372–385. 10.1111/psyp.12538 26877129 PMC4756390

[B82] Willis M. L. Palermo R. Burke D. Atkinson C. M. McArthur G. (2010). Switching associations between facial identity and emotional expression: a behavioural and ERP study. *Neuroimage* 50 329–339. 10.1016/j.neuroimage.2009.11.071 19962443

[B83] Wu Y. Zhou X. (2009). The P300 and reward valence, magnitude, and expectancy in outcome evaluation. *Brain Res.* 1286 114–122. 19539614 10.1016/j.brainres.2009.06.032

[B84] Yasuda A. Sato A. Miyawaki K. Kumano H. Kuboki T. (2004). Error-related negativity reflects detection of negative reward prediction error. *Neuroreport* 15 2561–2565. 15538196 10.1097/00001756-200411150-00027

[B85] Yeung N. Sanfey A. G. (2004). Independent coding of reward magnitude and valence in the human brain. *J. Neurosci.* 24 6258–6264. 15254080 10.1523/JNEUROSCI.4537-03.2004 PMC6729539

